# 1,25-Vitamin D-mediated hypercalcaemia in the setting of immune therapy-related sarcoid-like reaction

**DOI:** 10.1530/EDM-24-0116

**Published:** 2025-02-04

**Authors:** R K Dharmaputra, N Sheriff, S Ravichandran

**Affiliations:** ^1^ Gold Coast University Hospital Department of Endocrinology, Southport, Australia; ^2^ Gold Coast University Hospital Department of Medicine, Southport, Australia; ^3^ Royal Darwin Hospital Department of Medicine, Darwin, Australia

**Keywords:** calcium, vitamin D, calcitriol, sarcoidosis, hypercalcaemia

## Abstract

**Summary:**

We presented a case of a 72-year-old male with severe hypercalcaemia of 3.84 mmol/L following the second cycle of immunotherapy with ipilimumab and nivolumab in the setting of metastatic melanoma with known bone metastases. Further investigations demonstrated hilar lymphadenopathy, which was not present in previous imaging, and subsequent hypercalcaemia work-up demonstrated a significantly elevated serum calcitriol level as high as 429 pmol/L. A diagnosis of drug-induced sarcoid-like reactions or DISRs was made on the basis of hypercalcaemia and hilar lymphadenopathy following immunotherapy. Hypercalcaemia was effectively treated with intravenous fluids and medical therapy including a short course of subcutaneous calcitonin, a total of 120 mg of denosumab and oral prednisolone.

**Learning points:**

- DISRs are a rare complication of immunotherapy and may mimic metastases. A temporal relationship between commencement of therapy and progression on clinical imaging is important in making an accurate diagnosis.
- Calcitriol-mediated hypercalcaemia secondary to DISRs is an important differential diagnosis to hypercalcaemia of malignancy and should be considered in patients who have undergone immunotherapy.
- Prednisolone should be considered as the next line of treatment after fluid therapy in patients with calcitriol-mediated hypercalcaemia. Prednisolone and denosumab both reach maximum clinical efficacy between 7 and 10 days. Therefore, treatment administration should be spaced out by at least five days to avoid iatrogenic hypocalcaemia.

## Background

Hypercalcaemia is defined as a serum calcium level (corrected) greater than 2.6 mmol/L or greater than the upper limit reference range used in a laboratory (1). Approximately 90% of cases are due to primary hyperparathyroidism or hypercalcaemia of malignancy. In the setting of malignancy, the development of hypercalcaemia has been identified as a poor prognostic marker, with a 50% mortality at 30 days (2). There are multiple mechanisms found to cause hypercalcaemia in malignancy. These commonly include parathyroid hormone (PTH)-related protein (PTHrp) secreted from tumour cells, known as humoural hypercalcaemia of malignancy, local osteolytic metastases or high-serum 1,25-dihydroxyvitamin D (calcitriol) causing increased calcium absorption and bone resorption (1).

Immune checkpoint inhibitors (ICIs) have recently become a crucial component of cancer therapy, subsequently triggering a range of immunotherapy-related adverse effects (IRAEs), including hypercalcaemia due to drug-induced sarcoid-like reactions (DISRs). DISRs are defined as systemic granulomatous reactions that occur in relation to the initiation of offending agents (3). Clinically, DISRs and sarcoidosis both commonly present with bilateral hilar lymphadenopathy, cutaneous lesions, uveitis and hypercalcaemia (3), thereby posing difficulty in distinguishing one from another. Unlike sarcoidosis, the resolution of symptoms with cessation of the offending drug can favour a diagnosis of DISRs.

Calcitriol production is influenced by PTH, fibroblast growth factor 23 (FGF-23) and phosphate levels. The mechanism responsible for hypercalcaemia in granulomatous diseases, such as sarcoidosis or tuberculosis, occurs due to extrarenal calcitriol production independent of PTH by activated mononuclear cells in the lungs and lymph nodes. These are additionally more resistant to normal negative feedback of calcitriol production, thereby resulting in increased intestinal calcium absorption.

Whilst DISRs may present with minimal symptoms, the presence of hypercalcaemia prompts the need for immediate management. The current guidelines for hypercalcaemia of malignancy recommend the use of either denosumab or IV bisphosphonate as the initial therapy. However, between the two, treatment with denosumab is favoured. This recommendation is based on a study that demonstrated fewer skeletal-related effects (SREs), greater hypocalcaemia and reduced bone turnover markers with denosumab compared to bisphosphonates. SREs refer to the direct and indirect effects of malignancy or its treatment on bone, i.e., pathological fractures, spinal cord compression or skeletal pain. The effect of enhanced hypocalcaemia can be deemed beneficial in this setting as it may serve as an additional compensatory mechanism to prevent the consequences of sarcoidosis-related hypercalcaemia. Of note, denosumab is preferred additionally in the setting of chronic kidney disease due to no dose reduction requirement (4).

Whilst posing additional morbidity and mortality, the presence of IRAEs (e.g. DISRs) has been associated with greater immunological response and thereby suggests a beneficial outcome of the offending agent.

## Case presentation

M, a 72-year-old Caucasian male receiving combination ipilimumab–nivolumab immunotherapy for metastatic melanoma, was advised to present to the emergency by his oncologist due to severe hypercalcaemia. His serum corrected calcium at the time of presentation was 3.84 mmol/L, and this was confirmed by repeat blood collection at the hospital. Despite the severity of hypercalcaemia, he was well orientated and able to provide a complete medical history. He reported a few days history of polydipsia, polyuria, fatigue, dry mouth and worsening cough. There were no reports of constipation, bone pain or muscle weakness. Cardiac rhythm was normal without any evidence of QT shortening or other forms of arrhythmia on 12-lead ECG.

His medical history includes metastatic melanoma with BRAF V600E mutation and bilateral pulmonary and skeletal metastases. This was initially treated with dabrafenib–trametinib combination in December 2020 and radiotherapy to the lesion on his left knee. Subsequently, he developed a treatment-related interstitial nephritis and later ceased this treatment. He was subsequently commenced on ipilimumab–nivolumab combination immunotherapy in September of 2021 (2 months prior to presentation). His other relevant comorbid conditions include prostate cancer with pelvic metastasis diagnosed in 2013 that was currently under surveillance. Prior treatment of prostate cancer included external beam radiation therapy and androgen deprivation therapy (bicalutamide–leuprolide). Additional medical histories include pathological rib fracture, hypertension, dyslipidaemia, adrenal incidentaloma, asthma, gout and osteoarthritis with bilateral knee replacement.

Of note, there was no history of haematological malignancy or any autoimmune conditions. There was no recent travel history of exposure to tuberculosis. He denied taking any calcium or vitamin D supplements and was not on any regular medications other than esomeprazole and paracetamol.

M is a retired mental health nurse, currently living alone and independent with his self-care and activities of daily living. He consumed a vegan diet for ten years and remained invested in the optimisation of his health. In addition to an adequate oral intake for current high energy and protein requirements, he had regular iron infusions and B12 injections for ongoing deficiency-related anaemia.

## Investigation

Our patient presented with acute severe hypercalcaemia of 3.84 mmol/L, which was confirmed with repeat measurement at the hospital. In addition, he also experienced pre-renal acute kidney injury with significant volume depletion. Serum creatinine was measured at 186 mmol/L, with an estimated glomerular filtration rate (GFR) of 30 mL/min/1.73 m^2^ and urea of 20.2 mmol/L. Serum phosphate was also raised up to a level of 1.62 mmol/L.

Table 1 lists the full panel of investigations performed to investigate hypercalcaemia. Serum PTH was suppressed at 1.1 pmol/L (1.4–9.1 pmol/L). Thyroid hormone profile and serum protein electrophoresis were unremarkable. Serum 25-hydroxycholecalciferol was within the normal reference limit at 70 nmol/L. 1,25-dihydroxycholecalciferol (calcitriol) was measured using high-performance liquid chromatography–tandem mass spectrometry (LC-MS) method and was significantly elevated at 429 pmol/L. The elevated serum corrected calcium paired with a high serum phosphate is consistent with calcitriol excess. Exogenous calcium and vitamin D ingestion were excluded on arrival. Given the history of prostate cancer, serum prostate specific antigen (PSA) level was measured and was not elevated, PTHrp was undetectable.

**Table 1:** Laboratory investigations at initial presentation.

Test	Values	Reference range
Ionised calcium		
Corrected calcium, mmol/L	3.85	2.10–2.60
Phosphate, mmol/L	1.62	0.75–1.50
Albumin, g/L	45	36–47
Serum creatinine, μmol/L	186	60–110
Urea, mmol/L	20.2	3.5–9.5
eGFR, mL/min/1.73 m^2^	30	
PTH, pmol/L	1.1	1.4–9.1
PTHrp, pmol/L	<1	<1.05
25-hydroxycholecalciferol, nmol/L	82	50–150
1,25-dihydroxycholecalciferol, pmol/L	429	48–190
Serum ACE level, U/L	118	37–221
TFT	Euthyroid	
ALP, U/L	84	30–110
ALT, U/L	8	<50
GGT, U/L	24	<61
Urinary Ca:Cr, mol/mol cr	1.4	0.04–0.45
Sputum Ziehl–Neelsen stain	Negative	
Serum protein electrophoresis	No monoclonal protein	
PSA, μg/L	0.35	

PTH, parathyroid hormone; TFT, thyroid function test.

The differential diagnosis for non-PTH-dependent hypercalcaemia is broad. In this setting, important differentials include humoural hypercalcaemia of malignancy, 1,25-dihydroxycholecalciferol or calcitriol-dependent hypercalcaemia and hypercalcaemia from malignancy-associated osteolytic lesions. Elevated serum phosphate is more consistent calcitriol-mediated hypercalcaemia.

**Figure 1:**
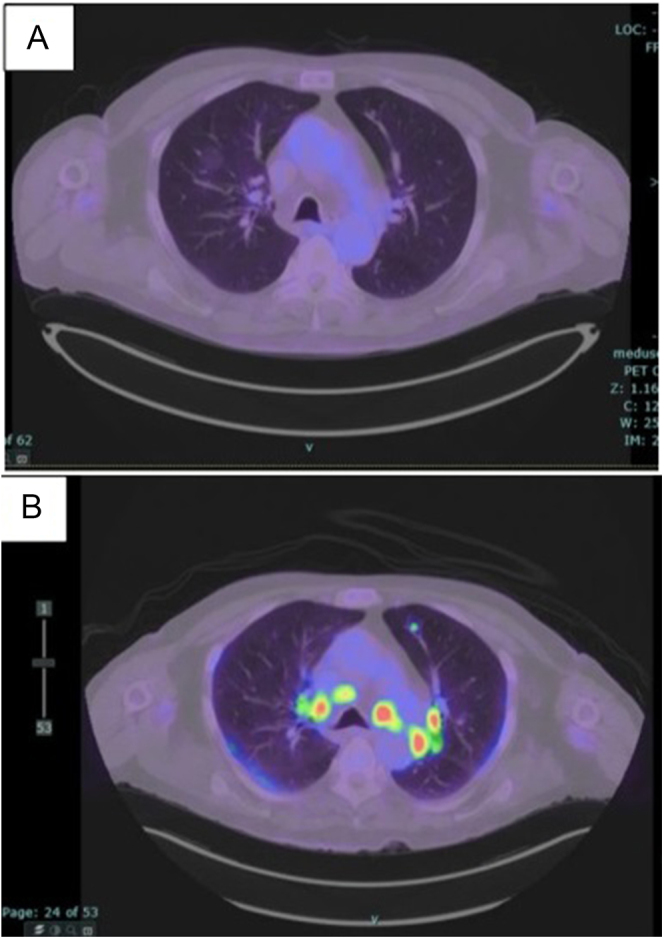
Axial CT/PET (A) before immunotherapy and (B) after immunotherapy.

In relation to radiological investigations performed throughout the medical admission, chest X-ray demonstrated a reduced size of pulmonary nodules and an increased size of hilar lymph nodes. A repeat PET scan further demonstrated treatment response to combined immunotherapy and an increased size of hilar lymph nodes as demonstrated in Fig. 1A and B. Ultrasound-KUB did not detect any kidney stone or nephrocalcinosis.

We made the diagnosis of severe hypercalcaemia secondary to DISRs based on the radiological finding of hilar lymphadenopathy and significant elevation in serum calcitriol following the second round of immunotherapy with ipilimumab and nivolumab. Of note, Ziehl–Neelsen stain was negative, and serum angiotensin converting enzyme (ACE) was measured at 118 U/L, which was within the normal reference range. Other rare causes for calcitriol-mediated hypercalcaemia include lymphoma, milk-alkali syndrome and exogenous vitamin D. The sarcoid-like reaction in our patient was limited to hilar lymphadenopathy, and hypercalcaemia, there were no other system involvements. Sarcoidosis and other granulomatous conditions increase the activity of 1α-hydroxylase enzyme, which converts 25-hydroxycholecalciferol to 1,25-dihydroxycholecalciferol (calcitriol). Calcitriol excess subsequently leads to increased absorption of both calcium and phosphate from the gastrointestinal tract, whilst also reducing renal excretion by increasing reabsorption of calcium and phosphate from the proximal convoluted tubule.

## Treatment, outcome and follow-up

**Figure 2:**
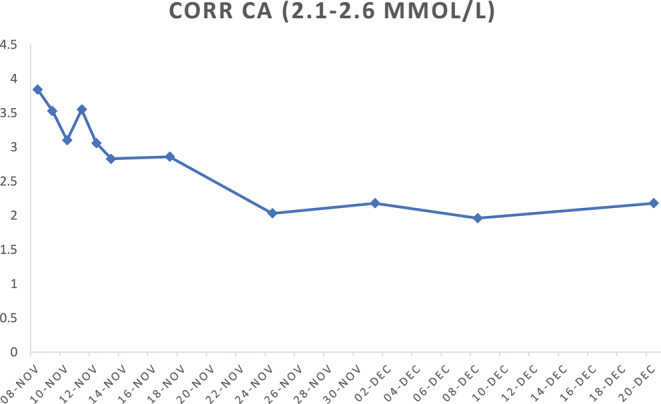
Corrected calcium trend in response to treatment.

Figure 2 illustrates the changes in serum corrected calcium levels following treatment. Intravenous fluid therapy with normal saline was commenced at the time of presentation. Four litres were infused over a 24-h period, which resulted in a modest reduction in serum corrected calcium from 3.84 to 3.53 mmol/L. Hypercalcaemia persisted despite aggressive fluid resuscitation; 60 mg subcutaneous denosumab were administered as adjunctive therapy and 100 IU subcutaneous calcitonin 6-hourly, which was limited to 24 h of use.

Glucocorticoid treatment with 50 mg oral prednisolone was commenced following the diagnosis of calcitriol-mediated hypercalcaemia and after active pulmonary tuberculosis had been ruled out. M was discharged to the community on prednisolone with a rapid steroid weaning plan. This, however, resulted in rebound hypercalcaemia up to 3.55 mmol/L, which had prompted a second admission for IV fluid therapy and a second dose of 60 mg denosumab. Prednisolone was increased back to 50 mg with a weekly weaning plan. Serum corrected calcium was 2.6 mmol/L at the time of hospital discharge, five days following the administration of the second dose of denosumab. Nine days following glucocorticoid therapy, serum calcitriol concentration declined from 429 to 65 pmol/L.

M had an ongoing drop in his serum corrected calcium in the community as low as 1.96 mmol/L whilst on treatment with prednisolone, which prompted the commencement of treatment with oral calcium replacement on day ten following treatment.

**Figure 3:**
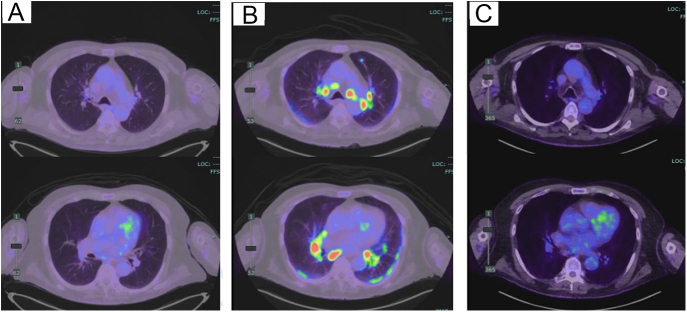
Axial CT/PET (A) before immunotherapy, (B) after immunotherapy and (C) after prednisolone.

Figure 3A, B, C illustrates the progression of PET imaging of the patient’s hilar lymph nodes before immunotherapy, after immunotherapy and following treatment with systemic glucocorticoid, respectively.

M was commenced on nivolumab monotherapy 6 months following the medical admission for hypercalcaemia and completed one cycle without complications. His treatment was later changed to encorafenib and binimetinib for 5 months due to disease progression. There was complete resolution of mediastinal lymphadenopathy on PET scan 6 months post-discharge. Unfortunately, the new treatment was poorly tolerated, and he later underwent palliative care and passed away.

## Discussion

We presented a unique case of hypercalcaemia in the setting of underlying malignancy with a range of differential diagnoses. In the setting of metastatic melanoma with bone involvement, the differential diagnosis in our patient includes humoural hypercalcaemia of malignancy, bone metastases-associated hypercalcaemia and calcitriol-mediated hypercalcaemia. Whilst hypercalcaemia in the setting of malignancy has been recognised as a negative prognostic marker, hypercalcaemia associated with DISRs secondary to immunotherapy may not carry the same prognostic ramification.

The current Endocrine Society Guidelines for hypercalcaemia of malignancy recommended the use of denosumab as a second-line treatment agent following IV hydration. Whilst denosumab has been proven to be effective in treating hypercalcaemia of malignancy, there is also a slightly higher rate of treatment-related hypocalcaemia as observed in our case (4). There are currently no specific guidelines for the treatment of calcitriol-mediated hypercalcaemia. Worldwide practices may therefore vary in relation to the use and timing of anti-resorptive and glucocorticoid therapy. In our case, we administered denosumab before prednisolone due to the delayed availability of the 1,25-vitamin D test result, which had enabled us to revise the patient’s diagnosis from hypercalcaemia of malignancy to DISRs.

Glucocorticoid therapy with high-dose prednisolone is the preferred treatment for calcitriol-mediated hypercalcaemia in the setting of granulomatous disease. Glucocorticoid directly inhibits the action of 1-alpha-hydroxylase in the activated mononuclear cells in the lungs and lymph nodes and therefore, reducing the conversion of 25-hydroxycholecalciferol to 1,25-dihydroxycholecalciferol or calcitriol. Furthermore, glucocorticoids also antagonise the action of calcitriol by inhibiting calcium absorption for the gastrointestinal tract (5). The net effect is that of a reduced serum calcium and calcitriol concentration within 2–5 days of administration, with a full effect in 7–10 days (6). There are currently little to no data on the concurrent use of an anti-resorptive agent and glucocorticoid in the setting of calcitriol-mediated hypercalcaemia. Our case has demonstrated that concurrent use of denosumab in this setting is highly effective and may lead to hypocalcaemia. Denosumab was initially prescribed due to the presumptive diagnosis of humoural hypercalcaemia of malignancy, and prednisolone was later added following the revision of diagnosis to calcitriol-mediated hypercalcaemia when serum calcitriol level became available. Therefore, in the setting of calcitriol-mediated hypercalcaemia secondary to granulomatous condition, glucocorticoid therapy should be considered second-line following IV fluid as it directly targets the pathophysiology of the underlying condition. Anti-resorptive treatment with bisphosphonate or denosumab should be considered in the setting of persistent hypercalcaemia despite high-dose glucocorticoid at least five days following initiation of glucocorticoid treatment to avoid hypocalcaemia secondary to over-treatment.

A DISR is a systemic granulomatous response, often occurring with the initiation of a causative agent. Drugs that have been commonly associated with this response include ICIs, anti-retroviral therapy, interferons and tumour necrosis factor-α antagonists. As the exact pathogenesis remains unknown, it is unclear if these drugs cause immunosuppression, exacerbating subclinical cases of true sarcoidosis or rather a separate entity altogether.

The most common ICI associated with DISRs is ipilimumab; however, there has been cases seen with nivolumab and pembrolizumab use. DISRs develop between three weeks and two years after the initiation of treatment. However, the majority of DISRs were diagnosed at an average of 4.6 months after the initiation of an ICI (3). A retrospective study of patients treated with ICIs demonstrated that DISRs occurred in 3.7 and 6.3% of patients treated with monotherapy and combination immunotherapy, respectively (7).

In the current literature, there have been similar reported cases of hypercalcaemia and granulomatous disease in patients following immunotherapy. A review of 26 case reports of DISRs due to ICIs revealed that the therapy was withheld in 38% of patients, 44% treated with systemic glucocorticoids and 8% with localised therapy (8). A comparable case involves a patient presenting with hypercalcaemia (ionised calcium 1.77) and only radiological evidence of DISRs following combination treatment for metastatic melanoma (9). As a result of symptomatic hypercalcaemia, their ICI was discontinued. This case highlighted that although DISRs are often asymptomatic and not life-threatening, the resultant hypercalcaemia can cause a range of complications prompting the need for immediate treatment and consideration of withdrawal of offending agents, like our reported case.

In conclusion, the growing field of immunotherapy highlights the importance of a sound understanding of its unique toxicity profile. The development of drug-induced sarcoid-like reactions following use of immune checkpoint inhibitors is of importance as it may clinically and radiologically mimic disease progression. Prompt recognition of this toxicity is crucial to initiate appropriate management of associated hypercalcaemia and measure therapeutic response to treatment.

## Declaration of interest

The authors declare that there is no conflict of interest that could be perceived as prejudicing the impartiality of the work.

## Funding

This work did not receive any specific grant from any funding agency in the public, commercial or not-for-profit sector.

## Patient consent

Written consent for publication of the article along with associated images was obtained directly from the patient before the commencement of writing.

## Author contribution statement

Each co-author has equal contribution in the writing of the case report. The lead author of the case report was directly involved in managing the patient.
